# Clinical Features of COVID-19, Dengue, and Influenza among Adults Presenting to Emergency Departments and Urgent Care Clinics—Puerto Rico, 2012–2021

**DOI:** 10.4269/ajtmh.22-0149

**Published:** 2022-11-21

**Authors:** Joshua M. Wong, Hannah R. Volkman, Laura E. Adams, Carene Oliveras García, Alma Martinez-Quiñones, Janice Perez-Padilla, Jorge Bertrán-Pasarell, Diego Sainz de la Peña, Rafael Tosado-Acevedo, Gilberto A. Santiago, Jorge L. Muñoz-Jordán, Brenda C. Torres-Velásquez, Olga Lorenzi, Liliana Sánchez-González, Vanessa Rivera-Amill, Gabriela Paz-Bailey

**Affiliations:** ^1^ Centers for Disease Control and Prevention, San Juan, Puerto Rico;; ^2^ Ponce Health Sciences University/Ponce Research Institute, Ponce, Puerto Rico;; ^3^ Auxilio Mutuo Hospital, San Juan, Puerto Rico

## Abstract

Dengue and influenza are pathogens of global concern and cause febrile illness similar to COVID-19. We analyzed data from an enhanced surveillance system operating from three emergency departments and an urgent care clinic in Puerto Rico to identify clinical features predictive of influenza or dengue compared with COVID-19. Participants with fever or respiratory symptoms and aged ≥18 years enrolled May 2012–January 2021 with dengue, influenza, or SARS-CoV-2 confirmed by reverse transcriptase polymerase chain reaction were included. We calculated adjusted odds ratios (aORs) and 95% CIs using logistic regression to assess clinical characteristics of participants with COVID-19 compared to those with dengue or influenza, adjusting for age, subregion, and days from illness onset to presentation for clinical care. Among 13,431 participants, we identified 2,643 with dengue (*N* = 303), influenza (*N* = 2,064), or COVID-19 (*N* = 276). We found differences in days from onset to presentation among influenza (2 days [interquartile range: 1–3]), dengue (3 days [2–4]), and COVID-19 cases (4 days [2–7]; *P* < 0.001). Cough (aOR: 0.12 [95% CI: 0.07–0.19]) and shortness of breath (0.18 [0.08–0.44]) were less common in dengue compared with COVID-19. Facial flushing (20.6 [9.8–43.5]) and thrombocytopenia (24.4 [13.3–45.0]) were more common in dengue. Runny nose was more common in influenza compared with COVID-19 (8.3 [5.8–12.1]). In summary, cough, shortness of breath, facial flushing, and thrombocytopenia helped distinguish between dengue and COVID-19. Although few features distinguished influenza from COVID-19, presentation > 4 days after symptom onset suggests COVID-19. These findings may assist clinicians making time-sensitive decisions regarding triage, isolation, and management while awaiting pathogen-specific testing.

## INTRODUCTION

Since the beginning of the COVID-19 pandemic, distinguishing between COVID-19 and other infectious diseases with overlapping signs or symptoms has presented a major diagnostic challenge for healthcare providers. Influenza and dengue are of particular concern in Puerto Rico and other dengue-endemic areas, as both cause nonspecific acute febrile illness and have discrete periods of high incidence.^1^ Although factors such as public health guidance for testing based on local disease activity will influence a healthcare provider’s clinical reasoning and management plan, most decisions made during the initial evaluation of a patient such as infection control measures or empiric management are based on patient history and clinical features that distinguish one pathogen from another.^2^^,^^3^

Dengue is the most common arboviral disease worldwide, with an estimated 390 million infections annually.^4^ Although dengue is commonly perceived in many endemic areas as a childhood disease,^5^ it also affects adults and can have distinct clinical manifestations compared with pediatric dengue, leading to missed diagnoses.^6^ Multiple reports of delayed identification of COVID-19 or dengue cases due to an incorrect initial clinical diagnosis^7^^,^^8^ highlight the need for studies comparing the natural history of these diseases and increased access to reliable testing.^3^^,^^9^^,^^10^

Influenza has been associated with 290,000–650,000 annual deaths worldwide,^11^ corresponding to ∼2% of all annual respiratory deaths before the COVID-19 pandemic.^12^ Both influenza and COVID-19 infect the respiratory tract, spread from person to person and were noted early in the pandemic to share many similarities in clinical presentation and transmission patterns.^13^ In Puerto Rico, seasonal influenza trends have synchronized with the influenza season in the US.^14^ Although studies comparing clinical features at presentation for influenza and COVID-19 in both adults^15^ and children^16^ have described the similarities in presentation between the two pathogens, laboratory confirmation remains key for diagnosis and management decisions such as isolation duration or empiric management, including appropriate antivirals and supportive care.^17^^,^^18^

We conducted an analysis comparing presenting clinical features of laboratory-confirmed influenza or dengue cases to COVID-19 among adults with a recent history of fever or respiratory symptoms enrolled in an enhanced surveillance system from multiple emergency departments, May 2012–January 2021, in Puerto Rico.

## MATERIALS AND METHODS

### Ethics statement.

The Institutional Review Boards at the CDC, Auxilio Mutuo, and Ponce Medical School Foundation approved the Sentinel Enhanced Dengue Surveillance System (SEDSS) study protocols 7301 and 6214, respectively.

### SEDSS patient population and study setting.

SEDSS was started in May 2012 and has enrolled patients with acute febrile illnesses from four healthcare facilities, including two emergency departments in tertiary care hospitals, one emergency department in a community hospital, and one urgent care clinic (Supplemental Table 1).

### Study enrollment and procedures.

Patients presenting to the emergency department (ED) were eligible for enrollment if they were febrile (oral temperature ≥38°C, axillary temperature ≥38.5°C) or reported a subjective fever within the last 7 days. Cough or shortness of breath within the last 14 days prior to presentation (with or without fever) was added as an eligibility criterion in April 2020. As previously described, recruiters administered a survey of self-reported symptoms of current illness, exposure history, and underlying conditions. Recruiters reviewed medical records from participants to capture vital signs, physical examination findings, laboratory findings, and disposition.^19^

### Diagnostic testing and definitions.

At enrollment, participants provided blood, nasopharyngeal, and oropharyngeal specimens for diagnostic testing. Serum samples were tested for dengue virus (DENV) by reverse transcription–polymerase chain reaction (RT-PCR) for DENV-1–4 and by IgM antibody capture (MAC)-ELISA for anti-DENV antibodies, as previously described.^19^ Nasopharyngeal/oropharyngeal samples were tested for influenza A and B, respiratory syncytial virus, parainfluenza virus 1 and 3, adenovirus, and human metapneumovirus using an RT-PCR–based respiratory viral panel as previously described,^19^ and for SARS-CoV-2, the virus that causes COVID-19, using an RT-PCR–based CDC assay.^20^ Dengue cases were defined as participants with DENV-1–4 detected by RT-PCR. Participants with a positive DENV IgM without DENV-1–4 detected by RT-PCR do not meet laboratory criteria for a confirmed case according to the 2015 dengue case definition^21^ and were not included in this analysis. Influenza cases were defined as participants with influenza A or B detected by RT-PCR. COVID-19 cases were defined as participants with SARS-CoV-2 detected by RT-PCR. Coinfections were excluded from this analysis.

### Data analysis.

We included participants ≥18 years old and calculated frequencies for sex, age, underlying conditions, disposition, and day of presentation for clinical care after illness onset (day 0 was considered the first day of illness onset) by pathogen. Using the clinical variables as the predictors and the disease as the outcome variable, we calculated odds ratios (ORs) for clinical and laboratory features at the time of presentation to the ED of dengue and influenza compared with COVID-19 cases, the referent group. We considered an adjusted OR (aOR) of >7.00 or 0.00–0.14 (the reciprocal) as a strong association, 2.00–7.00 or 0.14–0.50 as a moderate association, and 1.00–2.00 or 0.50–1.00 as a weak association.^22^ If the range of the 95% CI of the aOR included 1, we considered it a nonspecific clinical characteristic. We categorized clinical characteristics as high frequency if found in 80–100% of participants with that disease, medium frequency if found in 40–79% of participants, and low frequency if found in 0–39% of participants.

Differences in proportions were tested by applying a χ^2^ test or a Fisher’s exact test when the cell size was ≤5. Medians for continuous variables were compared using the Mann–Whitney–Wilcoxon test for two variables or the Kruskal–Wallis test for three or more variables. All analyses were done in SAS v. 9.4 (SAS Institute Inc., Cary, NC).

We performed stepwise selection and tests for interaction of all variables that could plausibly confound the relationship between exposures and outcome. Likelihood ratio χ^2^ calculations and the Hosmer–Lemeshow goodness-of-fit test for calibration were applied to parsimoniously construct the adjusted logistic regression models and select variables for the adjusted models. Area under the receiver operating characteristic curve values were used to measure model discrimination. Adjusted ORs were used to control for age, days from symptom onset to presentation, and subregion of the study enrollment site.

## RESULTS

A total of 13,431 adult participants were enrolled in SEDSS from May 7, 2012 to January 31, 2021. We included 2,643 cases in our analysis, consisting of 2,064 influenza cases (78%), 303 dengue cases (12%), and 276 COVID-19 cases (10%) (Supplemental Figure 1). Among the 2,064 influenza cases, there were 1,577 influenza A and 487 influenza B cases. Among the 303 dengue cases, there were 277 DENV-1 (91%), 2 DENV-2 (1%), 0 DENV-3, and 23 DENV-4 (8%) cases, and 1 case with an unspecified subtype (<1%). The first participants with COVID-19 in the analysis became ill in March 2020. At that time, the circulating SARS-CoV-2 had a wide diversity of B.1x lineages. In August 2020, the autochthonous lineage B.1.588 emerged in Puerto Rico and became the predominant lineage until the end of the analysis period.^23^

### Participant demographics.

The median age of the participants differed among the three diseases assessed (*P* < 0.001) (Table 1). Participants with COVID-19 were the oldest with a median age of 50 years (interquartile range [IQR] 35–59), followed by influenza with a median age of 38 years (IQR 26–54). Participants with dengue had the lowest median age at 30 years (IQR 22–47).

**Table 1:** Characteristics of adult participants presenting to the emergency department and urgent care clinic with COVID-19, dengue, or influenza; Sentinel Enhanced Dengue Surveillance System—Puerto Rico, 2012–2021

Characteristic	COVID-19 (*N* = 276)	Dengue (*N* = 303)*	Influenza (*N* = 2,064)	*P* value
Sex				0.07
Male	127 (46)	157 (52)	925 (45)	
Female	149 (54)	146 (48)	1,139 (55)	
Age, years, median (IQR)	50 (35–59)	30 (22–47)	38 (26–54)	<0.001
Age range, years				<0.001
18–49	136 (49)	236 (78)	1,415 (69)	
50–64	97 (35)	39 (13)	384 (19)	
≥65	43 (16)	28 (9)	265 (13)	
Days from illness onset to presentation, median (IQR)	4 (2–7)	3 (2–4)	2 (1–3)	<0.001
Outcome†				<0.001
Discharged from ED	150 (65)	172 (60)	1,890 (92)	
Admitted or transferred	67 (29)	115 (40)	163 (8)	
Death	13 (6)	1 (<1)	4 (<1)	
Enrollment site				<0.001
San Juan metro area subregion				
Auxilio Mutuo Hospital	201 (73)	56 (18)	140 (7)	
Southern Puerto Rico subregion				
SLEH, Ponce	48 (17)	167 (55)	1,040 (50)	
CEMI, Ponce	27 (10)	1 (0.3)	686 (33)	
SLEH, Guayama	–	79 (26)	197 (10)	

Values are no. (%) unless otherwise indicated. Differences in proportions were tested by applying a χ^2^ test. If the cell size was ≤5, a Fisher’s exact test was used. Medians for continuous variables (i.e., age and days from illness onset to presentation) were compared using the Kruskal–Wallis test for three or more variables. CEMI = Centro de Emergencia y Medicina Integrada; IQR = interquartile range; SLEH = San Lucas Episcopal Hospital.

* We identified 19 cases with a positive DENV IgM without DENV-1–4 detected by RT-PCR, which were not included in this analysis (Supplemental Table 2). We identified two coinfections of DENV and influenza and one coinfection of DENV and SARS-CoV-2, which were excluded from the analysis.

† Admission and death were mutually exclusive; however, all participants who died were also admitted to the hospital.

### Underlying conditions.

Forty-four percent of participants with dengue had at least one underlying condition that was significantly less frequent compared with COVID-19 (78%; aOR 0.32; 95% CI 0.21–0.50). Sixty-three percent of participants with influenza had underlying conditions, which was significantly less common compared with COVID-19 (aOR 0.56 [95% CI 0.37–0.84]) (Table 2).

**Table 2:** Underlying conditions of adult participants presenting to the emergency department and urgent care clinic with COVID-19, dengue, or influenza; Sentinel Enhanced Dengue Surveillance System—Puerto Rico, 2012–2021

	COVID-19 (*N* = 276 [referent])	Dengue (*N* = 303)		Influenza (*N* = 2064)	
Underlying condition	No. (%)	No. (%)	Adjusted odds ratio (95% CI)*	No. (%)	Adjusted odds ratio (95% CI)†
At least one underlying condition	215 (78)	134 (44)	0.32 (0.21–0.50)	1,294 (63)	0.56 (0.37–0.84)
At least two underlying conditions	122 (44)	64 (21)	0.54 (0.35–0.85)	677 (33)	0.74 (0.51–1.07)
Obesity (BMI > 30)‡	119 (47)	49 (29)	0.53 (0.33–0.83)	693 (39)	0.73 (0.52–1.04)
Class III obesity (BMI > 40)‡	28 (11)	7 (4)	0.42 (0.17–1.02)	116 (7)	0.70 (0.38–1.26)
Asthma	56 (20)	43 (14)	0.75 (0.46–1.22)	361 (17)	1.04 (0.69–1.57)
Coronary heart disease‡	11 (4)	18 (6)	2.63 (1.08–6.38)	114 (6)	1.77 (0.79–3.94)
Diabetes‡	46 (17)	24 (8)	0.65 (0.35–1.21)	324 (16)	0.94 (0.59–1.52)
High blood pressure	92 (34)	47 (16)	0.72 (0.43–1.21)	504 (24)	0.85 (0.56–1.30)
High cholesterol	49 (18)	27 (9)	1.03 (0.56–1.87)	225 (11)	0.88 (0.54–1.43)
Cancer‡	16 (6)	5 (2)	0.93 (0.30–2.89)	55 (3)	0.92 (0.40–2.08)
COPD‡	3 (1)	1 (<1)	0.54 (0.05–5.70)	28 (1)	1.84 (0.44–7.70)
Immunodeficiency	5 (2)	3 (1)	0.83 (0.16–4.22)	15 (1)	0.55 (0.13–2.23)
Chronic kidney disease‡	8 (3)	5 (2)	1.47 (0.40–5.35)	21 (1)	0.64 (0.20–2.01)
Chronic liver disease	0	2 (1)	–	12 (1)	–
Sickle cell disease	0	1 (<1)	–	10 (<1)	–
Thyroid disease	37 (14)	17 (6)	0.53 (0.27–1.04)	211 (10)	0.77 (0.46–1.29)

BMI = body mass index; COPD = chronic obstructive pulmonary disease.

* Base adjustment models for dengue compared with COVID-19 included age, day of presentation after illness onset, and subregion and achieved strong discrimination with an area under the receiver operating characteristic curve of 0.91. Hosmer–Lemeshow goodness-of-fit tests were used to assess the model fit for logistic regression, and was *P* = 0.16.

† Adjusting for the same variables for influenza compared with COVID-19, the area under the receiver operating characteristic curve was 0.92. Hosmer–Lemeshow goodness-of-fit tests was *P* = 0.097.

‡ These conditions are identified as having a significant association with risk of severe COVID-19 illness in at least one meta-analysis or systematic review.^45^

### Days from symptom onset to presentation and outcome.

**Figure 1.**
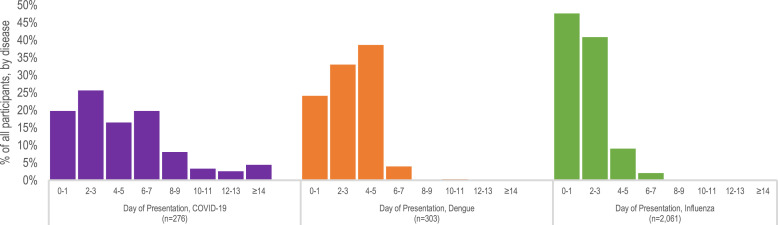
Percentage of all participants by disease and by day of presentation to emergency department or urgent care clinic for clinical care after illness onset; Sentinel Enhanced Dengue Surveillance System—Puerto Rico, 2012–2021.

We found significant differences in median day of presentation for clinical care after illness onset between participants with dengue compared with participants with COVID-19 (*P* < 0.001), and between participants with influenza compared with participants with COVID-19 (*P* < 0.001) (Table 1). Influenza cases had the shortest illness onset with a median of 2 days (IQR 1–3) followed by dengue cases with a median of 3 days (IQR 2–4). COVID-19 cases had a median of 4 days from symptom onset to presentation, with a right skew in its distribution (IQR 2–7 days) compared with the symmetric distribution of dengue or influenza (Figure 1).

We found differences in participant outcomes by disease (*P* < 0.001) with the highest mortality in participants with COVID-19 (6%) and lower mortality in those with dengue (<1%) and influenza (<1%) (Table 1).

#### Dengue versus COVID-19.

**Figure 2.**
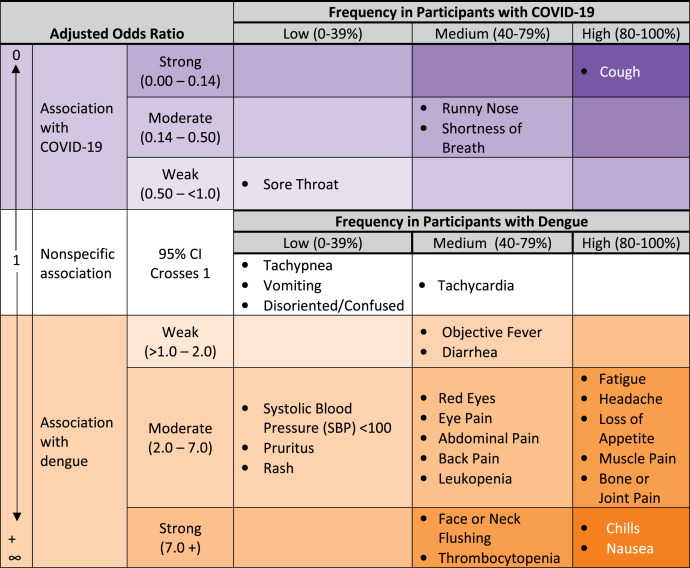
Association of symptoms, vital signs, and laboratory findings with dengue or COVID-19 among adult participants presenting to the emergency department with COVID-19, dengue, or influenza; Sentinel Enhanced Dengue Surveillance System—Puerto Rico, 2012–2021.

Cough was significantly less common among dengue cases (37%) compared with COVID-19 patients (aOR 0.12 [95% CI 0.07–0.19]), where it was found with high frequency (85%), resulting in a strong association with COVID-19 (Table 3, Figure 2). Runny nose was less frequently reported in dengue cases (24%) compared with COVID-19 cases, where it was found with medium frequency (41%), yielding a moderate association with COVID-19 (aOR 0.47 [95% CI 0.31–0.71]). Shortness of breath also had a moderate association with COVID-19 with significantly lower frequency in dengue cases (15%) compared with COVID-19 (56%; aOR 0.18 [95% CI 0.08–0.44]).

**Table 3:** Vital signs, symptoms, and clinical laboratory values at the time of presentation to the emergency department and urgent care clinic in adult participants with COVID-19, dengue, or influenza; Sentinel Enhanced Dengue Surveillance System—Puerto Rico, 2012–2021

	COVID-19 (*N* = 276 [referent])	Dengue (*N* = 303)		Influenza (*N* = 2,064)	
Clinical feature	No. (%)	No. (%)	Adjusted odds ratio (95% CI)*	No. (%)	Adjusted odds ratio (95% CI)†
Vital signs					
Objective fever‡	28 (14)	142 (49)	1.94 (1.14–3.30)	1,002 (49)	1.37 (0.84–2.22)
Tachycardia§	67 (25)	119 (41)	1.48 (0.97–2.26)	910 (45)	1.73 (1.18–2.52)
Tachypnea‖	48 (18)	54 (19)	1.46 (0.87–2.44)	187 (9)	0.77 (0.48–1.24)
Low SBP¶	3 (1)	21 (7)	4.86 (1.18–19.97)	89 (4)	3.01 (0.75–12.12)
Systemic					
Chills	174 (64)	271 (90)	7.11 (4.22–11.99)	1,766 (86)	5.73 (3.82–8.59)
Nausea	139 (51)	261 (87)	7.51 (4.72–11.94)	1,391 (68)	2.78 (1.96–3.95)
Vomiting	87 (32)	101 (34)	1.17 (0.77–1.76)	395 (19)	0.67 (0.46–0.97)
Fatigue	237 (87)	271 (91)	2.77 (1.50–5.10)	1,942 (94)	5.80 (3.42–9.83)
Headache	211 (78)	275 (91)	3.99 (2.28–7.01)	1,806 (88)	3.56 (2.29–5.53)
Loss of appetite	147 (54)	241 (81)	6.97 (4.44–10.96)	1,447 (70)	4.83 (3.32–7.03)
Pruritis	22 (8)	75 (25)	6.22 (3.39–11.44)	128 (6)	1.40 (0.77–2.55)
HEENT					
Red eyes	51 (19)	144 (49)	3.27 (2.13–5.02)	968 (47)	3.02 (2.04–4.48)
Eye pain	94 (35)	205 (69)	4.74 (3.15–7.13)	1,031 (50)	2.47 (1.74–3.53)
Runny nose	111 (41)	72 (24)	0.47 (0.31–0.71)	1,683 (82)	8.33 (5.75–12.06)
Sore throat	100 (37)	82 (28)	0.63 (0.42–0.94)	1,405 (69)	4.01 (2.83–5.70)
Pulmonary					
Cough	234 (85)	108 (37)	0.12 (0.07–0.19)	1,928 (94)	3.22 (1.99–5.19)
Shortness of breath	153 (56)	7 (15)	0.18 (0.08–0.44)	–	–
Gastrointestinal					
Diarrhea	99 (36)	124 (42)	1.78 (1.19–2.66)	453 (22)	0.88 (0.61–1.27)
Abdominal pain	91 (33)	166 (56)	2.86 (1.92–4.25)	683 (33)	1.3 (0.92–1.86)
Musculoskeletal					
Muscle pain	181 (67)	254 (85)	3.05 (1.92–4.84)	1,722 (84)	2.79 (1.91–4.08)
Bone or joint pain	153 (56)	249 (83)	3.79 (2.45–5.88)	1,717 (84)	3.77 (2.63–5.40)
Back pain	155 (58)	227 (77)	2.51 (1.66–3.81)	1,469 (72)	1.84 (1.30–2.59)
Integumentary					
Face or neck flushing	10 (4)	141 (47)	20.63 (9.79–43.48)	605 (29)	10.61 (5.10–22.09)
Rash	19 (7)	23 (38)	6.7 (3.15–14.26)	54 (4)	1 (0.50–2.01)
Neurologic					
Disoriented or confused	38 (14)	13 (22)	1.46 (0.69–3.10)	124 (9)	2.09 (1.24–3.52)
Laboratory findings					
Leukopenia#	44 (20)	166 (58)	5.51 (3.39–8.95)	138 (8)	0.44 (0.28–0.69)
Thrombocytopenia**	20 (9)	190 (67)	24.42 (13.26–44.99)	–	2.20 (1.23–3.93)
WBC, × 10^3^ cells/mm^3^, median (IQR)	5.6 (4.2–7.2)	3.5 (2.7–4.8)	–	6.5 (5.2–8.1)	–
Platelet count, × 10^3^/mm^3^, median (IQR)	215 (174–271)	114 (76–164)	–	198 (163–237)	–

Values are no. (%) unless otherwise indicated. HEENT = head, eyes, ear, nose, and throat; SBP = systolic blood pressure; WBC = white blood cell count.

* Base adjustment models included age, day of presentation after illness onset, and subregion and achieved strong discrimination with an area under the receiver operating characteristic curve of 0.91. Hosmer–Lemeshow goodness-of-fit tests were used to assess the model fit for logistic regression and was *P* = 0.16.

† Adjusting for the same variables, the area under the receiver operating characteristic curve was 0.92. Hosmer–Lemeshow goodness-of-fit tests was *P* = 0.097.

‡ Objective fever was only analyzed for participants reporting a subjective fever. It was defined as a temperature ≥ 38°C.

§ Tachycardia was defined as a heart rate > 100 beats per minute.

‖ Tachypnea was defined as a respiratory rate > 20 breaths per minute.

¶ Low SBP was defined as an SBP < 100 mm Hg.

# Leukopenia was defined as a WBC < 4,000 cells/mm^3^.

** Thrombocytopenia was defined as a platelet count of <150 × 10^3^/mm^3^.

Facial flushing was strongly associated (aOR 20.63 [95% CI 9.79–43.48]) with dengue (47%) and found with medium frequency compared with COVID-19 (4%). Thrombocytopenia was also found with medium frequency in dengue cases (67%) and low frequency in COVID-19 cases (9%) and was strongly associated with dengue (aOR 24.42 [95% CI 13.26–44.99]).

Most signs, symptoms, and clinical laboratory results were moderately associated with dengue compared with COVID-19 or were nonspecific for either dengue or COVID-19, including many considered characteristic of the acute phase of dengue (Table 3, Figure 2). Findings with high frequency in dengue cases and a moderate association with dengue compared with COVID-19 include headache, loss of appetite, bone or joint pain, and muscle pain. Red eyes, eye pain, leukopenia, and abdominal pain were found with medium frequency in dengue cases and moderately associated with dengue compared with COVID-19. Rash and pruritus were found with low frequency in dengue cases and had a moderate association with dengue.

#### Influenza versus COVID-19.

**Figure 3.**
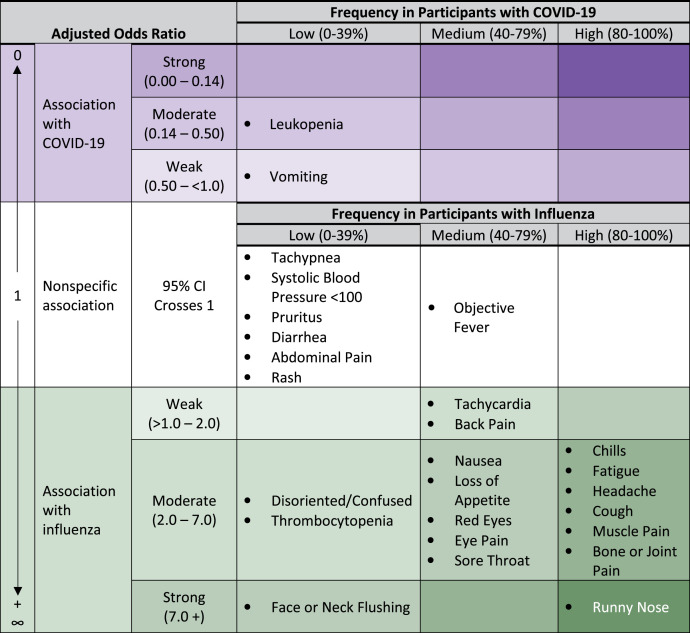
Association of symptoms, vital signs, and laboratory findings with influenza or COVID-19 among adult participants presenting to the emergency department with COVID-19, dengue, or influenza; Sentinel Enhanced Dengue Surveillance System – Puerto Rico, 2012–2021.

Overall, we found few clinical characteristics strongly associated with either COVID-19 or influenza. Leukopenia was less frequently reported in influenza cases (8%) compared with COVID-19 cases (aOR 0.44 [95% CI 0.28–0.69]) but was found with an overall low frequency (20%) in COVID-19 cases (Table 3, Figure 3).

Runny nose was found with high frequency in influenza cases (82%) compared with COVID-19 cases (41%) and was strongly associated (aOR 8.33 [95% CI 5.75–12.06]) with influenza. Face or neck flushing also had a strong association (aOR 10.61 [95% CI 5.10–22.09]) with influenza (29%) compared with COVID-19 (4%), although the frequency in influenza was low.

Most signs, symptoms, and clinical laboratory results were more common in, and moderately associated with, influenza compared with COVID-19 or nonspecific for either influenza or COVID-19 (Table 3, Figure 3).

## DISCUSSION

We found significant differences in median participant age, time from symptom onset to presentation, symptoms, signs, and laboratory findings between dengue and influenza cases compared with COVID-19 cases among adult participants enrolled in an enhanced surveillance system in Puerto Rico. For all three diseases, the time to presentation provided valuable information about the potential causative pathogen. Participants with influenza and dengue presented to medical care with a median of 2 and 3 days after symptom onset, respectively, with smaller interquartile ranges of ±1 day. COVID-19 had a longer median time to presentation of 4 days with a wider interquartile range of 2–7 days. A similarly longer time from symptom onset to presentation for care (analyzed as a binary of ≤3 days or >3 days) was predictive of COVID-19 compared with dengue or other febrile illnesses in a cohort study from Reunion Island.^24^ Another study from Switzerland comparing influenza and COVID-19 found that the time from symptom onset to presentation for care was a median of 3 days for influenza and 7 days for COVID-19.^25^ Our analysis reinforces observations from these previous studies comparing the time of presentation for clinical care of these three diseases.

Comparing dengue to COVID-19, the presence of both upper and lower respiratory symptoms (e.g., cough, shortness of breath) favored a laboratory diagnosis of COVID-19 as would be expected from a virus primarily infecting the respiratory tract. Similarly, a constellation of the musculoskeletal complaints characteristic of “breakbone fever” (dengue) and systemic complaints such as chills or nausea favored dengue. Facial flushing is considered a sensitive and specific marker of disease.^26^ We found a very strong association of facial or neck flushing with dengue compared with COVID-19, which, given this finding in about half of our dengue cases, could support its diagnosis if regularly elicited during the patient interview. Thrombocytopenia also had a strong association with dengue and was found in about two-thirds of the dengue cases in our analysis. Because platelet counts vary during the clinical course,^27^ serial collection of complete blood counts to detect falling platelet counts could be a distinguishing clinical finding for dengue and is already the standard of care in dengue clinical management.^28^ If recognized early, mortality and complications from dengue can be reduced to <1% with appropriate monitoring for warning signs that predict progression to severe disease and prompt initiation of a protocolized fluid management strategy,^28^^,^^29^ highlighting the importance of maintaining a high level of suspicion for dengue when evaluating undifferentiated febrile illness.

We found fewer symptoms and laboratory values that strongly favored influenza compared with COVID-19 than in the comparison of dengue to COVID-19. The only symptom strongly associated with influenza over COVID-19 and commonly found in influenza was runny nose. Our findings are consistent with a systematic review comparing symptoms in COVID-19 with other respiratory pathogens that found symptoms such as runny nose, sore throat, headache, cough, and myalgias more common in influenza versus COVID-19.^30^ Because CDC and Infectious Diseases Society of America guidelines recommend empiric antiviral treatment of patients with suspected or confirmed influenza at high-risk for complications,^18^ these findings could influence healthcare providers’ empiric treatment decisions. In practice, differentiating between influenza and COVID-19 based on clinical features will always be uncertain due to the multiple overlapping symptoms of these two respiratory tract infections.

While a diagnosis based on clinical findings can assist the first-line provider make a presumptive diagnosis, testing for DENV, influenza, and SARS-CoV-2 is key to workup and management in settings where these viruses are circulating. SARS-CoV-2 antigen tests are increasingly available worldwide, and their strategic use in resource-limited settings is an important tool in controlling the spread of COVID-19.^31^ Appropriate implementation and evaluation of these tests in combination with rapid diagnostic tests for DENV^9^ or influenza^32^ are urgently needed. However, improving laboratory infrastructure in dengue endemic areas to support definitive diagnosis with RT-PCR or validated antigen testing for both DENV and SARS-CoV-2 with results in a clinically meaningful timeframe should be a top priority both for improving local disease surveillance and clinical management.

Our study had several limitations. First, our inclusion criteria excluded cases of any of the three disease of interest that did not experience fever, cough, or shortness of breath. COVID-19, in particular, is less likely to present with fever than dengue or influenza.^33^ We were limited by the absence in SEDSS of several key findings that have been previously found to be predictive and clinically useful for diagnosing COVID-19 or influenza or evaluating their severity. These include new loss of taste or smell,^34^^,^^35^ chest pain,^35^ and productive cough^35^^,^^36^ as well as objective variables including oxygen saturation or chest examination findings^34^ and laboratory findings such as white blood cell differential (including absolute lymphocyte and neutrophil counts)^37^ or inflammatory markers.^38^ Although leukopenia has been associated with severe disease and higher mortality,^37^ our study enrolled all patients presenting to the ED resulting in a lower rate of mortality than the populations in these studies, likely explaining the lower frequency of leukopenia in our participants. New variants of SARS-CoV-2^39^ emerging after the study inclusion period as well as infections in vaccinated persons^40^ could also present with a different clinical phenotype from the COVID-19 cases included in this analysis. Oxygen saturation and shortness of breath were added to SEDSS in April 2020 and thus were not available to most participants with dengue and all participants with influenza, because most were enrolled prior to this modification (Supplemental Figure 1). Additionally, we only included dengue cases diagnosed by RT-PCR (confirmed cases), which is positive during the first week of illness, and did not include cases diagnosed by serology (probable cases), which is detectable later in the course of illness.^21^^,^^41^ However, our supplemental analysis of the characteristics of participants by the diagnostic method suggests that including only confirmed dengue cases instead of confirmed and probable dengue cases does not influence the day of presentation after illness onset (Supplemental Table 2). Lastly, we did not compare the clinical features of dengue to influenza in this analysis to focus our results on comparing two diseases with which healthcare providers have longstanding experience in diagnosis and management to COVID-19, a new disease with a rapidly evolving understanding of its clinical presentation.

If, even after a detailed exposure, travel, and immunization history, the causative pathogen remains unclear, our findings may assist in making time-sensitive clinical decisions related to triage, isolation, and empiric treatment in the absence of diagnostic test results. Our findings are of particular importance to providers practicing in jurisdictions where all three diseases circulate and where limited availability of diagnostic testing leaves clinical findings as the key to diagnostic reasoning.^3^ They are also useful for improving syndromic surveillance systems in these jurisdictions and to public health officials who incorporate this information into their decision-making and planning for these diseases. Vaccines against COVID-19, dengue, and influenza are licensed and currently recommended for use.^42^ Better surveillance for these diseases will aid in efforts to improve equity in access to and research on the impact of new vaccine tools.^43^ Although healthcare providers in resource-limited settings have quickly updated their clinical acumen to recognize and treat COVID-19, they must maintain a high clinical suspicion for dengue, influenza, and other viral causes of disease.^44^ Our findings highlight that the clinical features that distinguish COVID-19 from influenza or dengue are an important tool in this complex disease milieu.

## Supplemental files


Supplemental materials
