# Supplementary material for: Clinical Features of COVID-19, Dengue, and Influenza among Adults Presenting to Emergency Departments and Urgent Care Clinics—Puerto Rico, 2012–2021
Source: Am J Trop Med Hyg. 2022 Nov 21;108(1):107–14. doi: 10.4269/ajtmh.22-0149 (PMC9833087; doi:10.4269/ajtmh.22-0149)
Supplement: Supplementary file 1 [file tpmd220149.SD1.pdf]

Supplementary Table 1. Characteristics of the Sentinel Enhanced Dengue Surveillance System (SEDSS) enrollment sites; Puerto Rico, 2012–2021.

Facility Name	Subregion	Type of Healthcare Setting	No. of Beds[a]	Annual visits to the emergency department	Annual Admissions	Enrollment Period
Auxilio Mutuo Hospital	San Juan Metro Area	Tertiary-Care Teaching Hospital	497	33,439[b]	8,240[b]	November 2018 to present
SLEH, Ponce	Southern Puerto Rico	Tertiary-Care Teaching Hospital	326	41,550[c]	16,541[c]	May 2012 to present
CEMI, Ponce	Southern Puerto Rico	Freestanding Emergency Department	0	16,927[c]	3,000[c]	May 2012 to present
SLEH, Guayama	Southern Puerto Rico	Community Hospital	116	40,000[d]	6,000[d]	February 2013 to September 2015

Abbreviations: CEMI, Centro de Emergencias y Medicina Integrada; SLEH, San Lucas Episcopal Hospital.

[a]Data from American Hospital Directory for most recent cost report (https://www.ahd.com/states/hospital_PR.html)

[b]Data from personal communication with personnel at Auxilio Mutuo for the year 2019.

[c]Data from personal communication with personnel at SLEH for the year 2019.

[d]Data from personal communication with study staff for SEDSS at SLEH, Guayama site for the year 2012.

Supplementary Table 2. Characteristics of adult participants presenting to the emergency department with dengue by diagnostic; Sentinel Enhanced Dengue Surveillance System – Puerto Rico, 2012–2021

Characteristic	Only IgM positive, n = 19	DENV-1–4 RT-PCR positive, n=303[a]	p value
Sex			0.21
M	7 (37)	157	
F	12 (63)	146	
Age, y, median (IQR)	60 (48–71)	30 (22–70)	<0.001
Age range, y			<0.001
18–49	5	236	
50–64	8	39	
≥ 65	6	28	
Days from illness onset to presentation, d, median (IQR)	3 (2–4)	3 (2–4)	0.87
Days from illness onset to presentation range, d			
0–1	4 (21)	73 (24)	
2–3	7 (37)	100 (33)	
4–5	7 (37)	117 (39)	
6–7	1 (5)	12 (4)	
≥8	0	1 (<1)	
Outcome[b]			0.10
Discharged from ED	16 (84)	172 (60)	
Admitted or Transferred	3 (16)	115 (40)	
Death	0	1 (<1)	
Enrollment Site			
San Juan Metro Area			<0.001
Auxilio Mutuo Hospital	1 (5)	56 (18)	
Southern Puerto Rico			
SLEH, Ponce	12 (63)	167 (55)	
CEMI, Ponce	6 (32)	1 (0)	
SLEH, Guayama	0	79 (26)	

Values are no. (%) unless otherwise indicated. Differences in proportions were tested by applying chi-square test, or Fisher's Exact test if the cell size was ≤5. Medians for continuous variables were compared using the Kruskal-Wallis test for three or more variables. Abbreviations

CEMI, Centro de Emergencia y Medicina Integrada; d, days; IQR, interquartile range; SLEH, San Lucas Episcopal Hospital.

[a]Only participants with DENV-1–4 diagnosed by RT-PCR were considered a dengue case and included in the main analysis. Of these 303 DENV-1–4 RT-PCR positive dengue cases, 132 were also IgM positive, and 13 were IgM negative.

[b]Admission and death were mutually exclusive, however all participants who died were also admitted to the hospital.

Supplementary Figure 1. Epidemic curve of cases of dengue, influenza, and COVID-19 by date of onset in adult participants enrolled in the Sentinel Enhance Dengue Surveillance System (SEDSS), Puerto Rico, 2012–2021.

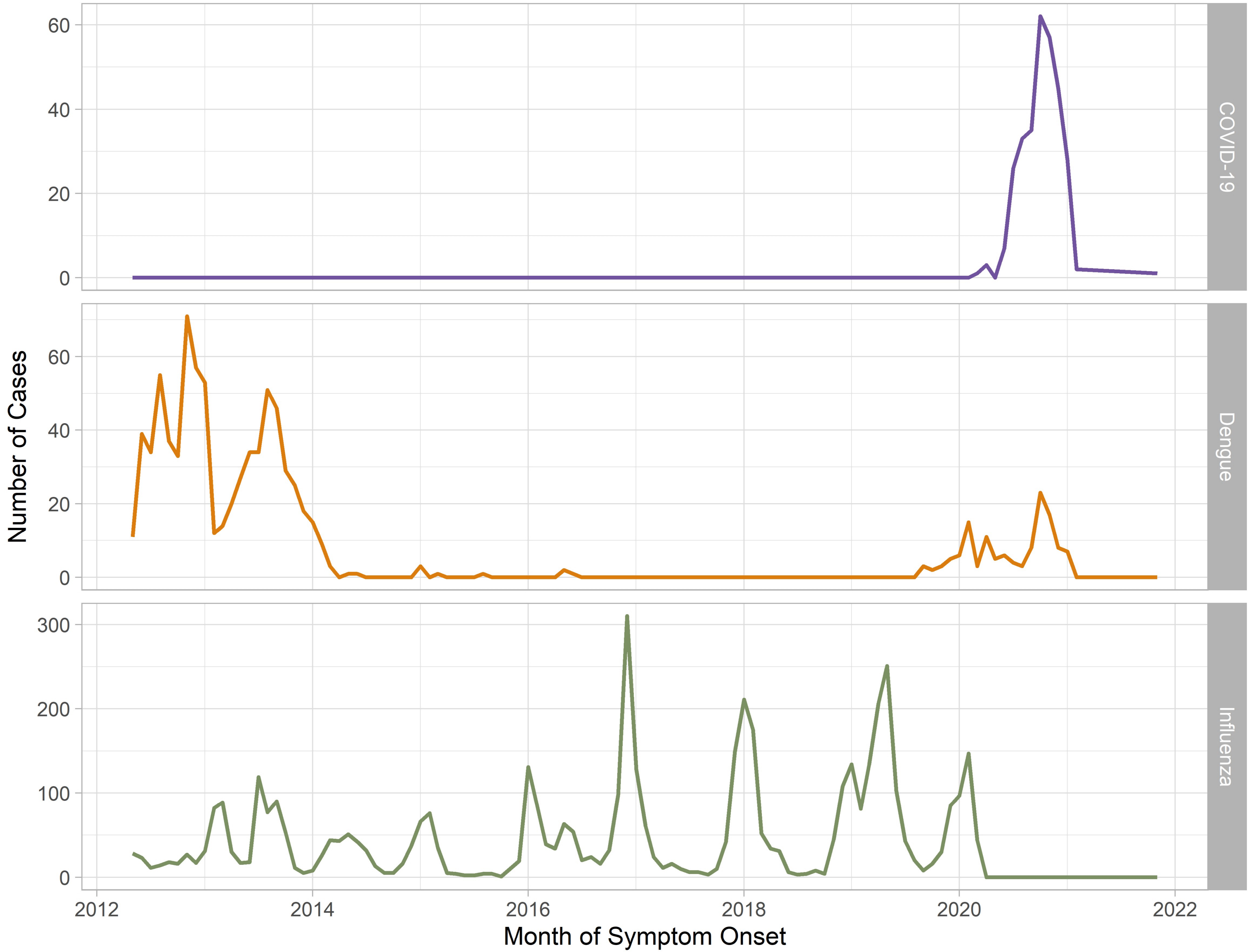

COVID-19
Dengue
Influenza
Number of Cases
Month of Symptom Onset
0
20
40
60
0
20
40
60
0
100
200
300
2012
2014
2016
2018
2020
2022
